# Identification of Three Elicitins and a Galactan-Based Complex Polysaccharide from a Concentrated Culture Filtrate of *Phytophthora infestans* Efficient against *Pectobacterium* *atrosepticum*

**DOI:** 10.3390/molecules191015374

**Published:** 2014-09-26

**Authors:** Guillaume Saubeau, Fanny Gaillard, Laurent Legentil, Caroline Nugier-Chauvin, Vincent Ferrières, Didier Andrivon, Florence Val

**Affiliations:** 1 INRA, UMR1349 IGEPP, Le Rheu-Cedex F-35653, France; E-Mail: didier.andrivon@rennes.inra.fr; 2 CNRS-Université Pierre et Marie Curie, FR2424, Station Biologique de Roscoff, Roscoff-Cedex 29682, France; E-Mail: gaillard@sb-roscoff.fr; 3 Ecole Nationale Supérieure de Chimie de Rennes, CNRS, UMR 6226, 11 Allée de Beaulieu, CS 50837, Rennes Cedex 7 35708, France; E-Mails: laurent.legentil@ensc-rennes.fr (L.L.); caroline.nugier@ensc-rennes.fr (C.N.-C.); vincent.ferrieres@ensc-rennes.fr (V.F.); 4 Agrocampus Ouest, UMR1349 IGEPP, Rennes F-35000, France; 5 Université Européenne de Bretagne, Rennes F-35000, France

**Keywords:** elicitin, GC-MS, MALDI TOF, PAMPs-triggered immunity, phenylalanine ammonialyase, polysaccharide, plant defense responses, *Solanum tuberosum*

## Abstract

The induction of plant immunity by Pathogen Associated Molecular Patterns (PAMPs) constitutes a powerful strategy for crop protection. PAMPs indeed induce general defense responses in plants and thus increase plant resistance to pathogens. *Phytophthora infestans* culture filtrates (CCFs) are known to induce defense responses and decrease the severity of soft rot due to *Pectobacterium atrosepticum* in potato tubers. The aim of this study was to identify and characterize the active compounds from *P. infestans* filtrate. The filtrate was fractionated by gel filtration, and the protection effects against *P. atrosepticum* and the ability to induce PAL activity were tested for each fraction. The fraction active in protection (F1) also induced PAL activity, as did the whole filtrate. Three elicitins (INF1, INF4 and INF5) were identified in F1b, subfraction of F1, by MALDI-TOF-MS and MS/MS analyses. However, deproteinized F1b still showed biological activity against the bacterium, revealing the presence of an additional active compound. GC-MS analyses of the deproteinized fraction highlighted the presence of a galactan-based complex polysaccharide. These experiments demonstrate that the biological activity of the CCF against *P. atrosepticum* results from a combined action of three elicitins and a complex polysaccharide, probably through the activation of general defense responses.

## 1. Introduction

*Pectobacterium atrosepticum*, formerly named *Erwinia carotovora* subsp. *atroseptica* [1], is a causal agent of bacterial soft rot and black leg, affecting both potato yield and tuber quality [2,3]. *P. atrosepticum*, a pectinolytic Gram negative bacterium, produces extracellular enzymes such as pectate-lyases, pectinases, cellulases and proteases, resulting in tissue maceration and rot symptoms [4] affecting postharvest storage [1] and causing significant economic losses ($20–100 million in multiple crops worldwide every year) [3,5,6]. 

There are currently no efficient curative methods to protect potato against *Pectobacterium* spp. [7], although biocontrol strategies have been tested to control soft rot development. For example, the application of biocontrol agents that are safe for the environment has become an important research area in pest management, and some fluorescent *Pseudomonas* strains can act as biological control agents against *Pectobacterium* spp. on melon [8] and on potato [9]. Fu *et al.* [10] found that a strain of *Bacillus subtilis* has an antagonistic control of the banana leaf spot disease in the field and the anthracnose disease at post-harvest stage of about 48.3% and 48.6% respectively. *Rhodococcus* bacteria can prevent disease due to *P. atrosepticum* by disrupting the quorum sensing-based communication of *P. atrosepticum* [11]. But these methods are not always efficient enough, and new strategies exploiting plant immunity constitute major opportunities for improved crop protection. In 2006, Jones and Dangl [12] described a conceptual coevolution model (zigzag model) between plants and pathogens. They showed that specific plant receptors (PRRs) detect microbial/pathogen associated molecular patterns (MAMPs/PAMPs). These molecules induce general defenses responses in plants and trigger PAMP-triggered immunity (PTI). To protect plants against pathogens, the strategy therefore aims at enhancing host recognition capacities for potential pathogens, at boosting the executive arsenal of plant immunity and at interfering with virulence signals used by microbial pathogens [13]. In bacteria, lipopolysaccharides (LPS) are considered as PAMPs both because they are molecules essential to the growth and survival of bacteria [14], but also because they are involved in activation of the plant defense mechanisms [15,16]. Erbs and Newman [17], reviewing the role of the LPS and the peptidoglycanes in plant immunity, emphasized their involvement in the induction of both early (burst oxidative, nitric, ionic flux) and later (induction of PR proteins, callose, and phenolic compounds) defense reactions. PAMPs are also found in fungal culture filtrates. Chalfoun *et al.* [18] showed that strawberry plants treated with a culture filtrate derived from an avirulent isolate of *Colletotrichum fragariae* prior to the inoculation with a virulent isolate developed significantly less disease. The disease was completely suppressed when plants were pre-treated 7 days before the challenge inoculation with the virulent isolate M11. The protection effect was due to the induction of defense responses (accumulation of reactive oxygen species and deposition of lignin and callose). The authors also showed that the response observed was cultivar-nonspecific. In the same way, a concentrated culture filtrate (CCF) of *Phytophthora infestans* (oomycete, causal agent of potato late blight) primed defense reactions in cell suspensions of potato cv. Bintje [19]. CCF also induced lipoxygenase (LOX) activity and the accumulation of various oxylipins in tobacco and potato cell suspensions [20], and phenylalanine ammonia-lyase (PAL) activity in tubers of different potato cultivars [21].

We have previously shown that CCF decreased the severity of soft rot caused by *P. atrosepticum* in potato tubers [19]. However, the precise composition of CCF remains unexplored, and hence the nature of active compounds. Since an innovative strategy, in crop management, is the use of purified natural or chemical compounds that enhance resistance plants against pathogens by inducing PTI, this information is crucial for an optimal use of eliciting properties of such filtrates. Yangui *et al.* [22] showed that soft rot development during potato tuber storage could be significantly reduced by treatment with a hydroxytyrosol-rich extract prepared from fresh olive mill wastewater. Natural plant volatiles can also control the potato postharvest disease due to *P. atrosepticum* [23].

The objectives of this study are therefore to: (i) identify and characterize the active compounds present in CCF; (ii) test their efficiency against *P. atrosepticum* in biotests and (iii) check their elicitor activity using PAL as a marker. After fractionation of CCF on gel filtration column and analyses of compounds by MALDI TOF and MS/MS, we demonstrated the implication of three elicitins and a galactan-based polysaccharide in the biological activity of culture filtrate. 

## 2. Results and Discussion

**Figure 1:**
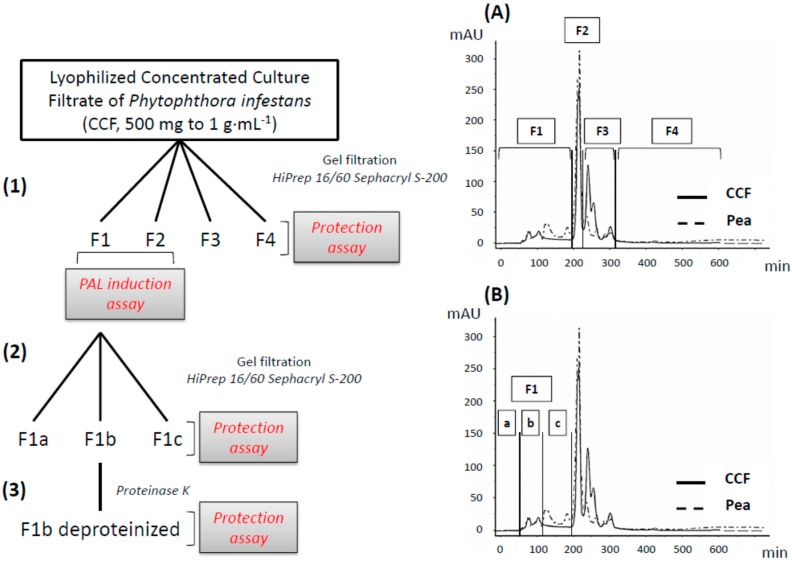
(**1**) The CCF is fractionated by gel filtration into four fractions (chromatogram (**A**) F1, F2, F3, and F4) and the induction of PAL activity is evaluated on F1 and F2 fractions. (**2**) The active fraction (F1) is separated by gel filtration into three subfractions (chromatogram (**B**) F1a, F1b and F1c). (**3**) F1b fraction was then deproteinized by the proteinase K. Each fraction (400 µg/slice) was tested on potato tubers for their protection effect against *P. atrosepticum*.

Four fractions (F1, F2, F3, and F4) and three sub-fractions of F1 (F1a, F1b and F1c) were obtained after gel filtration of crude CCF (Figure 1). These were tested for their ability to reduce soft rot symptoms in a tuber biotest, and the chemical composition of active fractions was analyzed.

### 2.1. Biological Activities of Crude and Fractionated CCF

#### 2.1.1. Protection of Potato Tubers against *P. atrosepticum*

##### Crude CCF

**Figure 2:**
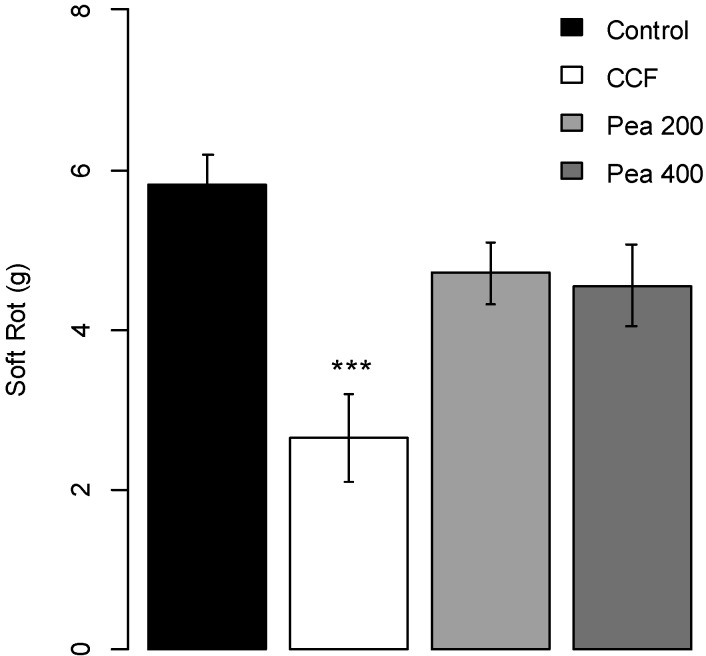
Protection effect of CCF (400 µg/slice) and pea (200 and 400 µg/slice) measured on potato tubers slices against *Pectobacterium atrosepticum*. Values are means of replicates of two independent experiments ± standard error. Bars with different stars subscripts are significantly different from control (water) (Tukey HSD, *** *p* = 0.001).

CCF at 400 µg/slice, but not pure pea broth used for growing the pathogen, significantly reduced (*p*-value = 0.0001) the soft rot severity due to *P. atrosepticum* compared to the water control (about 40%, Figure 2). Disease severity on potato tubers treated with CCF at 50, 100 or 200 µg/slice were lower, but not statistically different, from that on the controls (*p*-values = 0.4454, 0.5674 and 0.2245 respectively, Supplementary Data Figure S1).

**Figure 3:**
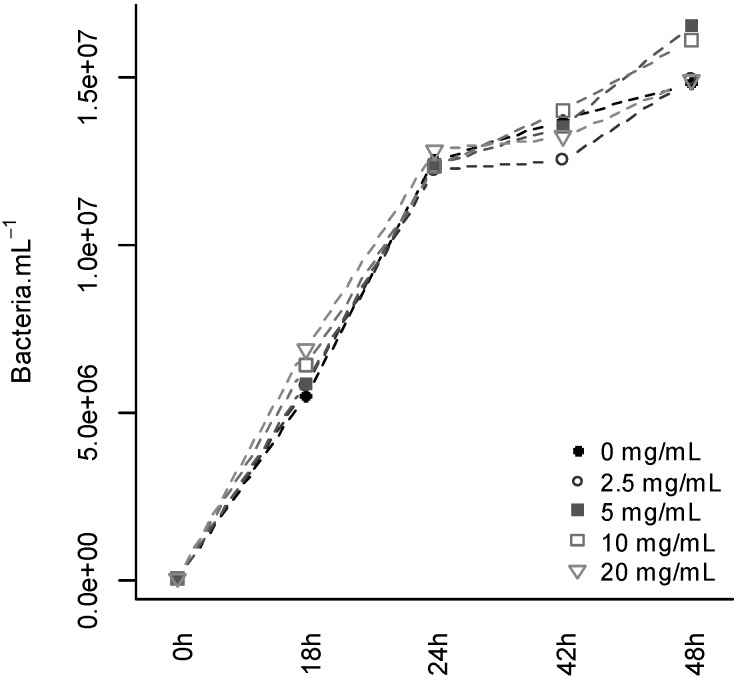
Effect of CCF on *Pectobacterium atrosepticum* growth *in vitro*. Concentration of *P. atrosepticum* measured 0 h, 18 h, 24 h, 42 h and 48 h of culture in liquid media enriched with different concentrations of CCF (0, 2.5, 5, 10 or 20 mg·mL^−1^).

After 48 h of culture, CCF (2.5, 5, 10 and 20 mg·mL^−1^) had no effect on *P. atrosepticum* growth (Figure 3). Since the two highest concentrations exceeded that used in the tuber slice biotest (equivalent to 8 mg·mL^−1^), the protection activity of CCF cannot be attributed to a direct toxic activity of the filtrate molecules on *P. atrosepticum* cells.

##### Protection Effect of Fractions

**Figure 4:**
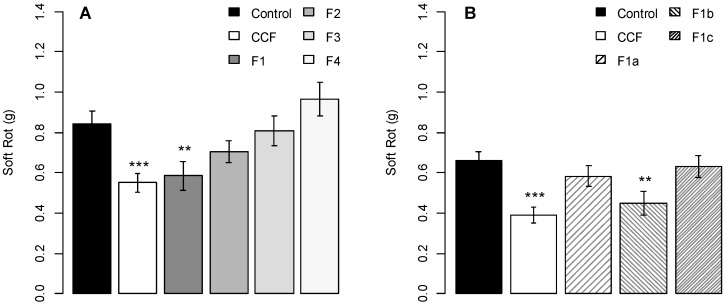
Protection activity of fractionated CCF (**A**) and of F1 sub-fractions (**B**) on potato tubers slices against *Pectobacterium atrosepticum*. Values are means of replicates of three independent experiments ± standard error. Bars with different stars subscripts are significantly different from the water control (Tukey HSD, *** *p* = 0.001, ** *p* = 0.05).

Of the four fractions obtained after gel filtration on a HiPrep 16/60 Sephacryl S-200 High Resolution column, only fraction F1 induced a protection efficacy (about 30% disease reduction; *p*-value = 0.0299) similar to that of the crude filtrate (Figure 4A). None of the other three fractions had any significant protection activity compared to the control (*p*-values = 0.6099, 0.9994, 0.8384 respectively).

Subsequent fractionation of the active fraction F1 yielded three fractions (F1a, F1b and F1c) (Figure 1), all low UV-active and with only little overlaps with the corresponding fractions from pea broth. Only F1b (*p*-value = 0.039) induced the same protection effect as the crude filtrate (F1a, *p*-value = 0.8317; F1c, *p*-value = 0.9955; Figure 4B) in the tuber biotest.

The CCF at 8 mg·mL^−1^ (400 µg/slice) but not at lesser concentrations, protected potato tubers against *P. atrosepticum*. These results, in accordance with the results we have previously obtained [19], show that a threshold concentration of the active compound(s) is needed to induce the decrease of symptoms. The biological activity is restricted to a small fraction of CCF (F1b), and is not mediated by the pea broth culture medium. Furthermore, the fact that CCF (and hence its biologically active fraction F1b) did not have any bactericidal effect against *P. atrosepticum* suggests that its protection activity is not due to direct toxic inhibition of the pathogen, and may rather result from indirect action through defense activation in potato tubers. We therefore tested the elicitor activity of CCF and its active fractions, using PAL activity as a broad-range marker. Shree Prasad *et al.* [24] also found that a culture filtrate from the bacterium *Serratia marcescens* GSm01 had antiviral effect against yellow strain of Cucumber mosaic virus (CMV-Y) on tobacco plants. Bioactivity of fungal culture filtrates against root-knot nematode *Meloidogyne incognita* egg hatch and juvenile motility were also showed by Bhat and Wani [25]. Culture filtrate of a strain of *Streptomyces globisporus* was also shown to suppress the infection process of *Magnaporthe oryzae* on rice leaves by inhibiting conidial germination and reducing appressorial formation [26]. 

#### 2.1.2. PAL Activity Induction on Potato Tubers

**Figure 5:**
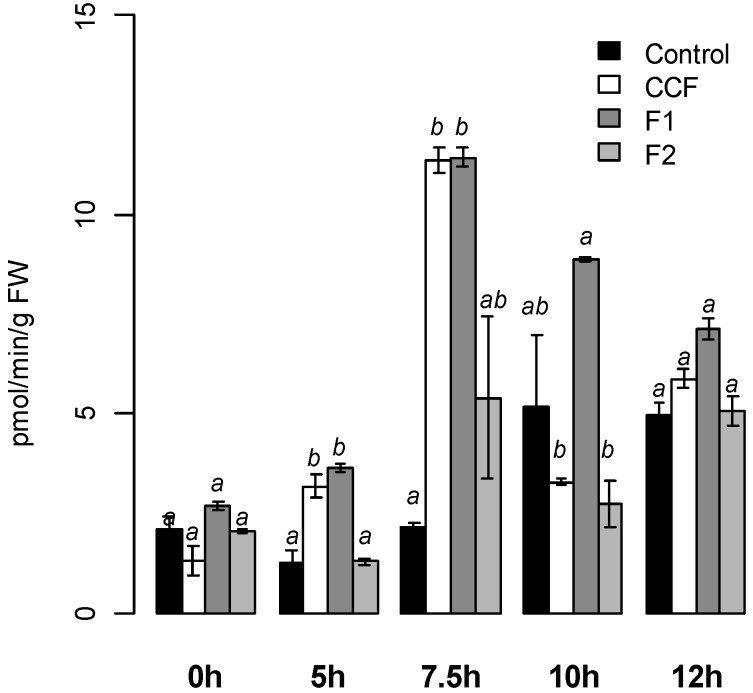
PAL activity measured on potato tubers slices after treatment with CCF, F1 or F2 (200 µg/mL). Values are means of two replicates of one experiment ± standard error. Bars with different letters subscripts are significantly different (Tukey HSD, *p* = 0.05).

Compared to the water control, CCF and F1 at 200 µg·mL^−1^ (Figure 5), but not F2 induced significantly PAL activity on potato tubers, with a maximum intensity achieved 7.5 h after treatment (12 pmol·min^−1^·g^−1^ FW, *p* < 0.03 and *p* < 0.02 respectively). PAL activity decreased after 10 h for CCF and 12 h for F1.

We previously showed that the CCF primes LOX induction in potato (cv. Bintje) cell suspensions [19], and triggers the oxylipin pathway in tobacco cell suspensions [20]. CCF also induces PAL activity in different genotypes of potato tubers [21]. PAL is a key enzyme of the phenylpropanoïd pathway, which leads to the synthesis of antimicrobial compounds, particularly chlorogenic acid. *In vitro*, chlorogenic acid decreases the growth of *P. atrosepticum* [27], suggesting that the protection effect was due to the induction of defense responses in potato tubers. In the same way, Bariya *et al.* [28] showed that a culture filtrate from *P. infestans* induces hypersensitive reactions on the leaves of potato genotypes resistant to this pathogen, but not of susceptible ones. Salicylic acid contents, phenylalanine ammonia lyase (PAL), β-1,3-glucanase and chitinase activities were induced in resistant genotypes [28].

### 2.2. Identification of Active Compounds

#### 2.2.1. Elicitins

**Figure 6:**
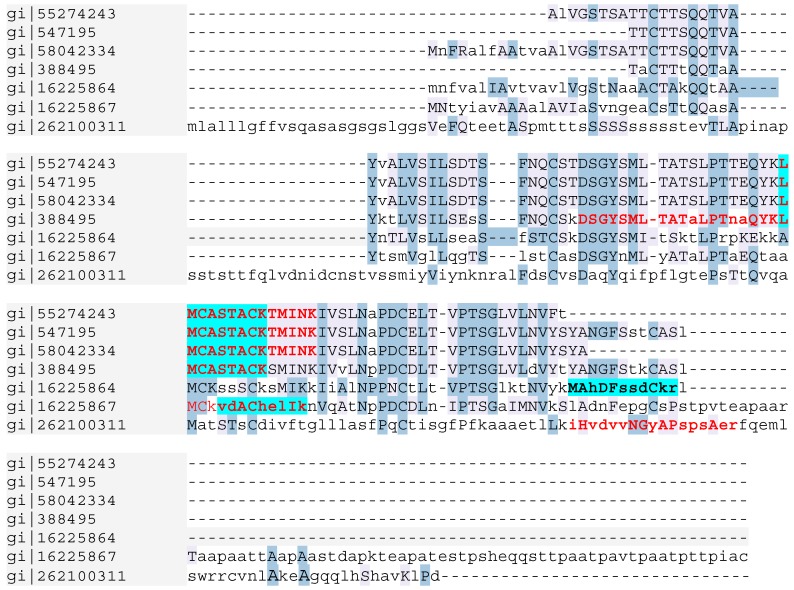
Alignment of peptides sequences obtained from MS/MS analysis (in bold) of F1b CCF active fraction (highlighted in blue) and crude CCF (in red) with elicitin peptide sequences in the NCBI database.

In the CCF, the MALDI TOF spectra revealed a massive peak with a 10,363.61 *m/z* value. In the F1 pea broth fraction (control), four different peaks with 12,145.66, 13,514.06, 13,675.30 and 13,835.31 *m/z* values were detected, suggesting that the corresponding proteins were degraded during *P. infestans* development (Supplementary data Figure S2A). The MS/MS analysis showed the CCF contained three peptides with a good sequencing score: LMCASTACKTMINK, DSGYSMLTATALPT NAQYK and VDACHELIK. Proteome Discoverer (Thermo Fisher Scientific) assigned the three peptides to the α-elicitin family. LMCASTACKTMINK and DSGYSMLTATALPTNAQYK sequences matched the same accession number: gi|55274243, corresponding to the elicitin precursor (INF1) after alignment in the NCBI database. VDACHELIK matched accession number gi|16225867, corresponding to elicitin-like INF5 from *P. infestans* in the NCBI database (Figure 6).

In the biologically active F1b subfraction, the MALDI TOF spectra (Supplementary Data Figure S2B) gave a massive peak with a 10,352.51 *m/z* value. The MS/MS analyses showed the presence of LMCASTACK and VDACHELIK peptides, belonging to the α-elicitin family as in CCF (Figure 6). A further peptide, MAHDFSSDCKR was only sequenced in the F1b fraction. After alignment, this peptide was found to match with the elicitin-like INF4 from *P. infestans* (accession gi|16225864). Several other peptides were detected by MS/MS in the CCF and F1b and corresponded to pea proteins, especially storage proteins of the seed (Table S1 in Supplementary Data).

**Figure 7:**
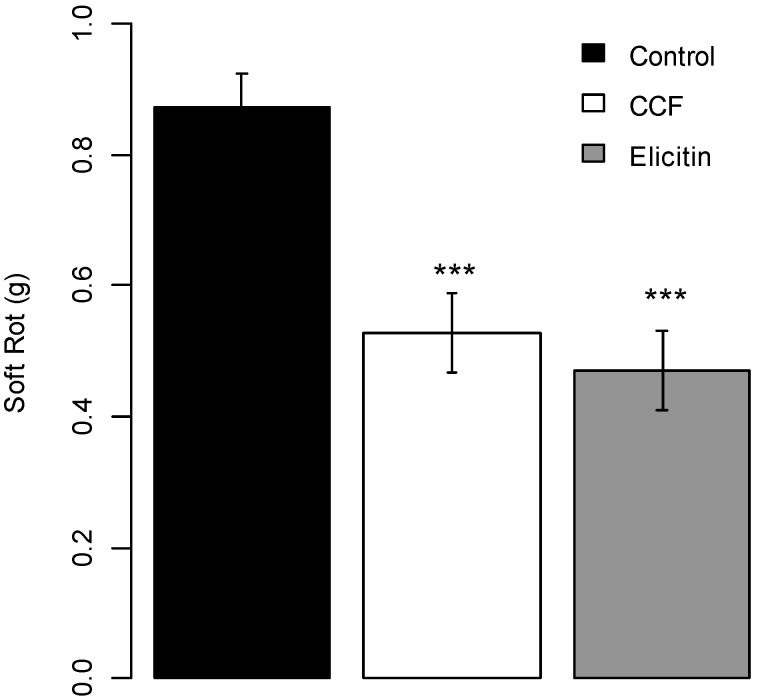
Protection effect of partially purified elicitin (400 µg/slice) on potato tubers slices against *Pectobacterium atrosepticum*. Values are means of replicates of one experiment with two blocks ± standard errors. Bars with different stars subscripts are significantly different from water control (Tukey HSD, *** *p* = 0.001, ** *p* = 0.05).

The elicitin fraction of CCF, partially purified on an exchange ion DEAE cellulose column and checked with MALDI TOF and MS/MS analyses for the presence of peptide sequences corresponding to INF4 and INF5 (Supplementary Data Figure S2C), gave a protection efficacy of tuber slices against *P. atrosepticum* equivalent to that the whole filtrate (*p*-value < 0.0001 compared to the control (Figure 7).

Our results thus showed that elicitins are part of the active fraction of CCF. Elicitins, low-molecular weight proteins secreted by Oomycete species [29], are a family of structurally related proteins with highly homologous sequences, which induce hypersensitive response in incompatible interactions. The *Phytophthora* elicitin family has been classified in two groups based of both structural properties and biological activities [29]. The α-elicitin group includes acidic proteins produced by *P. parasitica*, *P. capsici* and *P. infestans*, whereas β-elicitins are basic proteins produced by *P. cryptogea*, *P. cinnamomi* and *P. megasperma* [30]. In *P. infestans*, seven elicitin genes have been cloned (*inf1*, *inf2a*, *inf2b*, *inf3*, *inf4*, *inf5* and *inf6*) [31,32,33]. INF1 is the major elicitin secreted by *P. infestans*. INF1 recognition could be a component of non-host resistance of tobacco to *P. infestans* [32]. In *P. infestans*, *inf1*, *inf5* and *inf6*, are among the most abundantly expressed genes in mycelium. *Inf4* is expressed to a lesser extent in mycelium and zoospores and neither *inf4* nor *inf5* are expressed during infection [34]. Thus the biological activity of the CCF observed against *P. atrosepticum* could be due to the ability of elicitins to induce defense reponses *in planta*. Different authors showed that elicitin could induce defense responses: purified infestin from a culture filtrate of *P. infestans* was found to induce defense responses such as glucose oxidase, NADPH oxidase, superoxide dismutase, glutathione reductase, catalase and peroxidase enzymes in potato plants resistant to *P. infestans* [28].The well described cryptogein, isolated from *P. cryptogea*, acts as an elicitor on tobacco plants inducing hypersensitive response, the production of the phytoalexins, ethylene biosynthesis and formation of active oxygen species (AOS) [35]. Cryptogein and capsicein from *P. capsici* also promote defense responses in *Quercus suber* against *P. cinnamomi* infection [36]. 

#### 2.2.2. Polysaccharides

**Figure 8:**
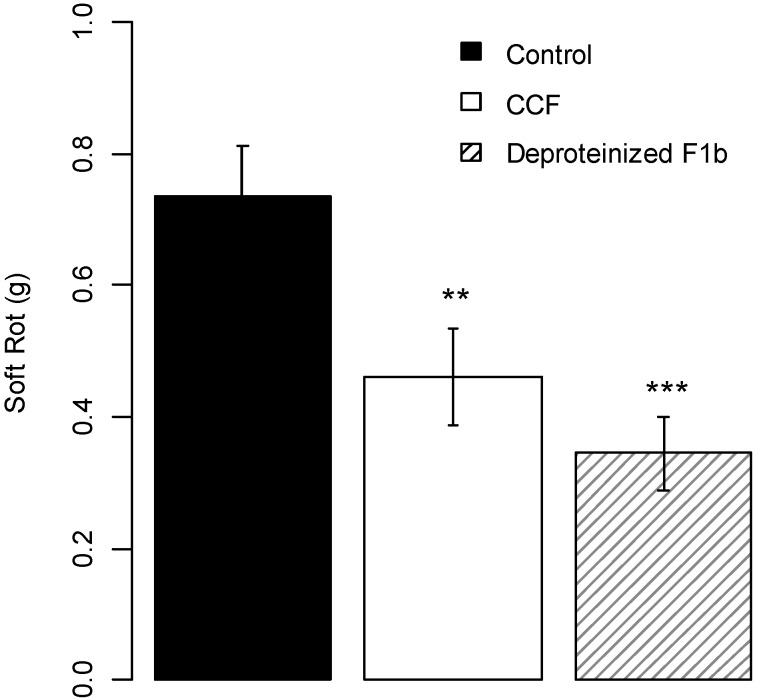
Protection effect of deproteinized F1b (400 µg/slice) on potato tubers slices against *Pectobacterium atrosepticum*. Values are means of replicates of two independent experiments with two blocks ± standard errors. Bars with different stars subscripts are significantly different from water control (Tukey HSD, *** *p* = 0.001, ** *p* = 0.05).

For lower *m/z* values (Supplementary Data Figure S2D), F1b treated with proteinase K showed a MS profile consistent with the presence of carbohydrates. To corroborate this hypothesis, the monosaccharide composition of CCF was established by gas chromatography (GC) through the itol composition obtained after the sequence acido-catalyzed hydrolysis/reduction with sodium borodeuteride/acetylation (Table 1). A similar procedure was also performed on pea broth. The main monosaccharides identified in CCF and pea broth are glucose, galactose and mannose, and to a lesser extent, arabinose, xylose and rhamnose. After DEAE chromatography, the GC data showed that the amount of glucose significantly decreased in both samples, and that amounts of other monosaccharides were similar or decreased, except that of galactose. Indeed, the F1b fraction from CCF showed increased presence of galactitol. Therefore, GC analysis showed the presence of a galactan-based, complex polysaccharide within fraction F1b. Deproteinised F1b had the same protection activity as the whole CCF (about 50% protection with *p*-value = 0.001 compared to the water control, Figure 8) suggesting that CCF contained active compound(s) other than elicitins.

**Table 1:** Molar ratio of itols identified in JDPP and CCF, and in the corresponding fractions isolated by chromatography (50–120 min).

Itol	Pea	F1b Pea	CCF	F1b CCF
Rhamnitol	0.1	5.4	0.2	0.0
Fucitol	0.0	6.1	0.0	0.0
Arabinitol	1.3	33.7	2.2	31.7
Xylitol	0.5	10.2	0.6	13.2
Mannitol	6.0	7.1	3.1	9.1
Galactitol	6.0	5.3	10.2	20.5
Glucitol	86.1	32.2	83.6	25.4

It is well known that oligo- and poly-saccharides can trigger defense responses in plants, enhancing protection against pathogens. Klarzynski *et al.* [37] showed that tobacco leaves infiltrated with laminarin (a linear β-1,3 glucan from the brown alga *Laminaria digitata*), had reduced symptoms of infection with soft rot pathogen. Laminarin was also shown to be both an efficient elicitor of defense responses in grapevine cells and plants. It effectively reduced *Botrytis cinerea* and *Plasmopara viticola* development on infected grapevine plants. Laminarin induced alkalinization of the extracellular medium of cell suspension cultures and a transient release of H_2_O_2_ [37,38]. Other oligosaccharides such as sulfated fucans, structural components of the cell walls of marine brown algae, induced a marked alkalinization of the extracellular medium of tobacco suspension cell cultures, the release of hydrogen peroxide, PAL and LOX activities [39]. Seaweed polysaccharides and derived oligosaccharides also trigger an initial oxidative burst at local level and the activation of phytohormone pathways (salicylic acid, jasmonic acid and/or ethylene) at systemic level, leading to an increased expression of genes encoding PR proteins with antifungal and antibacterial activities or other defense enzymes such as PAL and LOX [40]. Wolski *et al.* [41] showed that α-glucan from a non-pathogenic *Rhizoctonia* induces plant defense responses (β-1, 3-glucanase and chitinase activities, callose and lignine deposition) and protects potato sprouts against Rhizoctonia canker and dry rot. By contrast with glucans, there is little published evidence about eliciting activities of galactan-based polysaccharides [42]. Our results are therefore quite original, and warrant further analysis of the fine structure of the complex active polysaccharides within CCF.

## 3. Experimental Section

### 3.1. Biological Material, Inoculum and Elicitor Preparation

Potato tubers (*S. tuberosum* L., cv. Bintje) were provided by INRA Ploudaniel, France. They were first washed with water to eliminate the ground traces and then dipped in a water solution containing 80% ethanol (v/v) during 5 min. When washed and cleaned, tubers slices (1 cm thick) were cut and rinsed under water to eliminate starch.

Inoculum of *P. atrosepticum* (CFBP 5889, INRA Angers, France [43]) and inoculum of *P. infestans* (isolate 08-P15-12 from the IGEPP collection, France) was prepared as described by Kröner *et al.* [21]. Bacterial concentrations were adjusted with distilled water to about 5 × 10^8^ cfu·mL^−1^ and inoculum of *P. infestans* was adjusted to a final concentration of 5 × 10^4^ sporangia·mL^−1^ before liberation of zoospores at 4 °C during 2 h.

CCF of *P. infestans* (08-P15-12 isolate), was prepared as described earlier [16]. *P. infestans* was grown in sterile pea broth during 3 weeks. The culture broth was prepared by boiling 125 g of frozen peas in 1.2 L of distilled water, discarding the peas and autoclaving the broth for 20 min at 120 °C. The filtrate was obtained by separating the mycelium from the culture broth on 0.45 µm Whatman filter paper, and lyophilized before use.

### 3.2. Biological Activities

*Protection assays of fractions*. CCF, pea broth and each fraction obtained from the CCF were tested for their protection effect against *P. atrosepticum* on potato slices (1 cm thick). Slices were transferred to plastic trays containing moistened filter paper in the dark at 20 °C. Droplets (50 µL/slice) of mother solutions of CCF in water (1, 2, 4 or 8 mg·mL^−1^) were pipetted, and then spread onto the whole surface of the slice with a glass rod. CCF fractions were tested in a similar way, but from a mother solution at 8 mg·mL^−1^ and only 400 µg/slice for the different fractions tested. After 48 h incubation, 50 µL of a suspension of *P. atrosepticum* was deposited at the center of each slice, and soft rot was weighed after 72 h incubation at 20 °C in the dark.

*Bactericidal activity of CCF*. The bactericide effect of CCF at 2.5, 5, 10 and 20 mg·mL^−1^ was assessed *in vitro* on *P. atrosepticum* growth. Culture medium without CCF served as the control. Liquid King B medium containing 10^5^ bacteria mL^−1^ was enriched with adequate amounts of CCF and placed on a rotary shaker at 300 rpm. After 18 h, 24 h, 42 h and 48 h at ambient temperature, bacterial concentration was determined by measuring the optical density at 580 nm on a spectrophotometer as described by Desender *et al.* [16].

*PAL activity*. PAL activity was estimated by measuring the formation of cinnamic acid from phenylalanine at 290 nm on potato disks, as described by Kröner *et al.* [20]. PAL activity was expressed as pmol of *trans*-cinnamic acid produced per min and mg fresh weight (FW). The quantification of PAL activity was performed on two aliquots of one biological experiment.

### 3.3. Purification of CCF Compounds

CCF and pea broth (500 mg to 1 g·mL^−1^) were separated on a prepacked gel filtration HiPrep 16/60 Sephacryl S-200 high Resolution column (GE Healthcare Life Sciences, Uppsala, Sweden) on AKTA Purifier with water as solvent. Four fractions from CCF and pea broth (named F1, F2, F3 and F4) were then tested for their protection effect against *Pectobacterium atrosepticum* on potato slices. Fraction 1 from the CCF was further separated into three fractions (F1a, F1b and F1c) after another purification step and tested for protection activity.

Elicitin was partially purified from CCF on an anion Hiscreen Capto DEAE column. The column was rinsed with water, then equilibrated in sodium acetate 5 mM pH 6 (4 vol./phase vol.). To the concentrated culture filtrate, 5 mM of sodium acetate was added and the pH adjusted to 6 with acetic acid. Then the column was eluated with sodium acetate 50 mM pH 4 (adjusted with TFA). The column was rinsed with increasing concentrations of NaCl in sodium acetate 5 mM pH 6 (0.25 M, 0.5 M and 1 M). The elicitins were recovered from the acetate pH 4 fraction. This fraction was adjusted to pH 7 prior to extensive dialysis (6000 Da cut-off) against ultrapure water. The resulting dialyzed fraction was then freeze dried.

### 3.4. Characterization of Active Compounds

#### 3.4.1. MALDI-TOF MS

Analyses by MALDI-TOF MS were performed using a Voyager DE-STR MALDI-TOF mass spectrometer (AB Sciex, Foster City, CA, USA). Each sample powder (F1a, F1b, F1c, F2, Elicitin, pea broth, CCF) was first diluted with water and then 1 µL of this solution was mixed with:

2,5-Dihydroxybenzoic acid (20 mg/mL in 50% ACN, 0.1% TFA; Sigma-Aldrich, St. Louis, MO, USA) for sugar detection.

Sinapinic acid matrix (10 mg/mL in 30% ACN, 0.1% TFA; Sigma) for protein detection and spotted onto the MALDI target. 

For low masses sugar detection, spectra were acquired in positive ion both linear and reflector modes under 20 kV accelerating voltage and a mass range of 100–5000 Da. External calibration was performed using the calibration mixture Peptide mix 4 (Laser Bio Labs, Sophia-Antipolis, France).

For high masses sugar detection, spectra were acquired in positive ion linear mode under 25 kV accelerating voltage and a mass range of 5–250 kDa. External calibration was performed using the calibration mixture E composed of cytochrome C (12384), horse heart myoglobin (16950), trypsinogen (23980), subtilisin (27288), yeast enolase (93069).

For protein detection, spectra were acquired in positive ion linear mode under 25 kV accelerating voltage and a mass range of 5–25 kDa. External calibration was performed using the calibration mixture E. Each solution was also digested with bovine trypsin, desalted and mixed with alpha cyano 4 hydroxycinnamic acid (10 mg/mL in 50% ACN, 0.1% TFA; Sigma) for peptide detection and spotted onto the MALDI target. Spectra were acquired in positive ion reflector mode under 20 kV accelerating voltage and a mass range of 100–5000 Da. External calibration was performed using the calibration mixture Peptide mix 4 (Laser Bio Labs).

#### 3.4.2. ESI LTQ Orbitrap MS MS

The peptides mixtures were also analyzed by online nanoflow liquid chromatography tandem mass spectrometry (LC-MS/MS) on an EASY-nLC IITM system (Proxeon, Odense, Denmark) connected to the LTQ Orbitrap Discovery instrument (Thermo Fisher Scientific, Bremen, Germany). 2 μL of the peptide mixtures were concentrated onto the 2-cm pre-analytical column (100-μm inner diameter) packed with 5-μm C18 beads (Easy-column, Proxeon). They were separated in a 10-cm analytical column (75-μm inner diameter) packed with 3-μm C18 beads (Easy-column, Proxeon) with a 60-min gradient from 5% to 35% acetonitrile in 0.1% formic acid. The effluent from the HPLC column was directly electrosprayed into the mass spectrometer. The LTQ Orbitrap instrument was operated in data-dependent mode to automatically switch between full scan MS and MS/MS acquisition. Instrument control was through Tune 2.5.5 and Xcalibur 2.1. 

The survey full scan MS spectra (from *m*/*z* 400–2000) were acquired in the Orbitrap system with resolution *r* = 30,000 (after accumulation to a target value of 5e6 in the linear ion trap). The three most intense peptide ions with charge states ≥2 were sequentially isolated to a target value of 1e5 and fragmented in the HCD collision cell with normalized collision energy of 35%. The resulting fragments were detected in the Orbitrap system with resolution *r* = 7500. The ion selection threshold was 1000 counts for HCD, and the maximum allowed ion accumulation times were 500 ms for full scans and HCD. 

Standard mass spectrometric conditions for all experiments were: spray voltage, 1.7 kV; no sheath and auxiliary gas flow; heated capillary temperature, 200 °C; predictive automatic gain control (AGC) enabled.

Proteome Discoverer (Thermo Fisher Scientific, Bremen, Germany) and NCBI database were used to determine the identity of the proteins.

#### 3.4.3. Polysaccharide Analysis

The polysaccharide analysis was carried out after proteins were removed from fraction F1b by treatment with proteinase K. A 2.5 mL aliquot of a 10 mg·mL^−1^proteinase K solution, prepared in 10 mM phosphate buffer, pH 7.6, from lyophilized enzyme fixed on an inert substrate (Sigma, P9290), was added to 5 mg F1b fraction and incubated for 8 h at 37 °C. Polysaccharides composition was determined according to a hydrolysis/reduction/acetylation sequence followed by mass spectrometry analysis of the resulting deuteriated residues. The polymer was hydrolyzed with 1 mL of 2.5 M TFA at 100 °C during 2 h. The reactive was evaporated. Reduction of the monomers in alditols was performed with 1 mL of 1M sodium borodeuteride (NaBD_4_) freshly prepared in 2 M NH_4_OH. The reaction was stopped by adding acetic acid (dropwise until the disappearance of bubbles). After concentration under reduced pressure, the products were then acetylated with 200 µL 1-methylimidazole and 2 mL of acetic anhydride. After quenching by adding 5 mL of water, the alditol acetates were extracted with 1 mL dichloromethane, dried over magnesium sulfate and concentrated. The resulting compounds were dissolved in 100 µL methanol before analysis by GC. Injector: split mode split. Column: Supelco fused silica capillary (30 m × 0.20 mm I.D., film thickness: 0.20 µm). Separation was achieved with a temperature program of 190 °C for 4 min, then ramped at 4 °C·min^−1^ to 230 °C and held for 15 min, and a constant flow of 1 mL·min^−1^. The injector and detector temperatures were 250 °C and 300 °C, respectively.

### 3.5. Statistical Analyses

All statistical analyses were performed with the statistical software R GUI version 3.0.2. Analyses of variance were performed using the function ‘aov.’ Transformations were applied when necessary to approximate normality. Multiple comparisons of means were carried out using the Tukey HSD (Honestly Significant Difference) Test (*p* = 0.05 **, *p* = 0.01 ***).

## 4. Conclusions

We report here the chemical and biological characterization of the concentrated culture filtrate of a strain of *P. infestans* (08-P15-12), isolated from potato. Our results described that two components from a culture filtrate from *P. infestans* (CCF), namely a mix of 3 alpha-elicitins and a galactan-based complex polysaccharide, can protect potato against *P. atrosepticum*. Both are contained within a single fraction of CCF, which showed no bactericide activity. The significant disease reduction they provide (up to 40%–50% relative to a water control) likely results from the activation of plant defense responses. It will be interesting to verify if CCF or active compounds confer protection of potato against other pathogens, and activate the same or different defense pathways. This will require a more complete purification of each of the active molecules, and to determine the stoichiometry of the CCF composition. Elucidating the exact nature of each of the active compounds, and particularly of the intriguing galactan-based complex polysaccharide–uncommonly referred to in the defense activation literature-will open new ground for optimising the mix composition and improve biocontrol options against difficult to control bacterial plant pathogens. 

## Supplementary Materials

Supplementary materials can be accessed at: http://www.mdpi.com/1420-3049/19/10/15374/s1.

## Supplementary Files

Supplementary File 1
