# Supplementary material for: Identification of Three Elicitins and a Galactan-Based Complex Polysaccharide from a Concentrated Culture Filtrate of Phytophthora infestans Efficient against Pectobacterium atrosepticum
Source: Molecules. 2014 Sep 26;19(10):15374–90. doi: 10.3390/molecules191015374 (PMC6270706; doi:10.3390/molecules191015374)
Supplement: Supplementary File 1 [file molecules-19-15374-s001.pdf]

Supplementary Materials

Figure S1. Protection effect of CCF (50 µg, 100 µg or 200 µg/slice) or control (water) measured on potato tubers slices against *Pectobacterium atrosepticum*. Values are means of replicates of two independent experiment ± standard error.

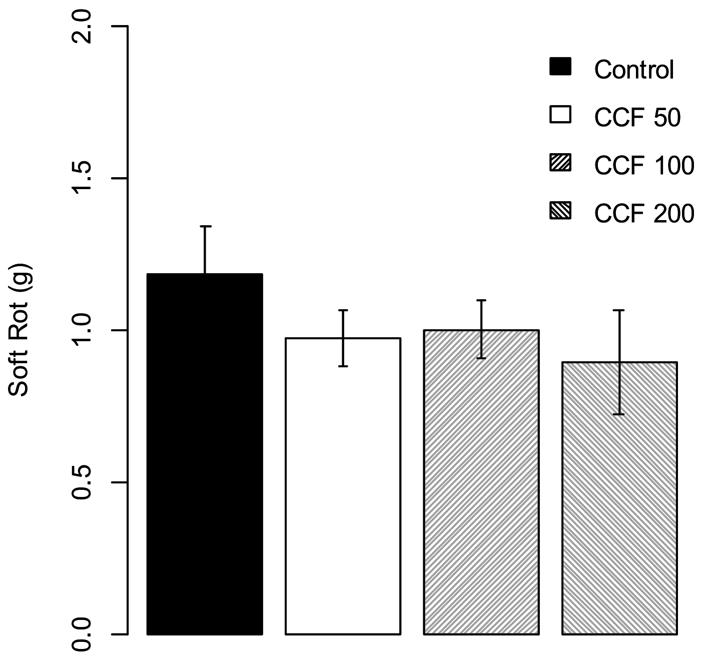

Figure S2. DHB mass spectra of (**A**) CCF (green), F1 from pea broth (red) and F2 from pea broth (gray); (**B**) DHB mass spectra of CCF (green) and F1b (red); (**C**) DHB mass spectra of CCF (red) and the partially purified elicitin (green). Mass spectrum of F1b between 200 and 2500 Da (**D**).

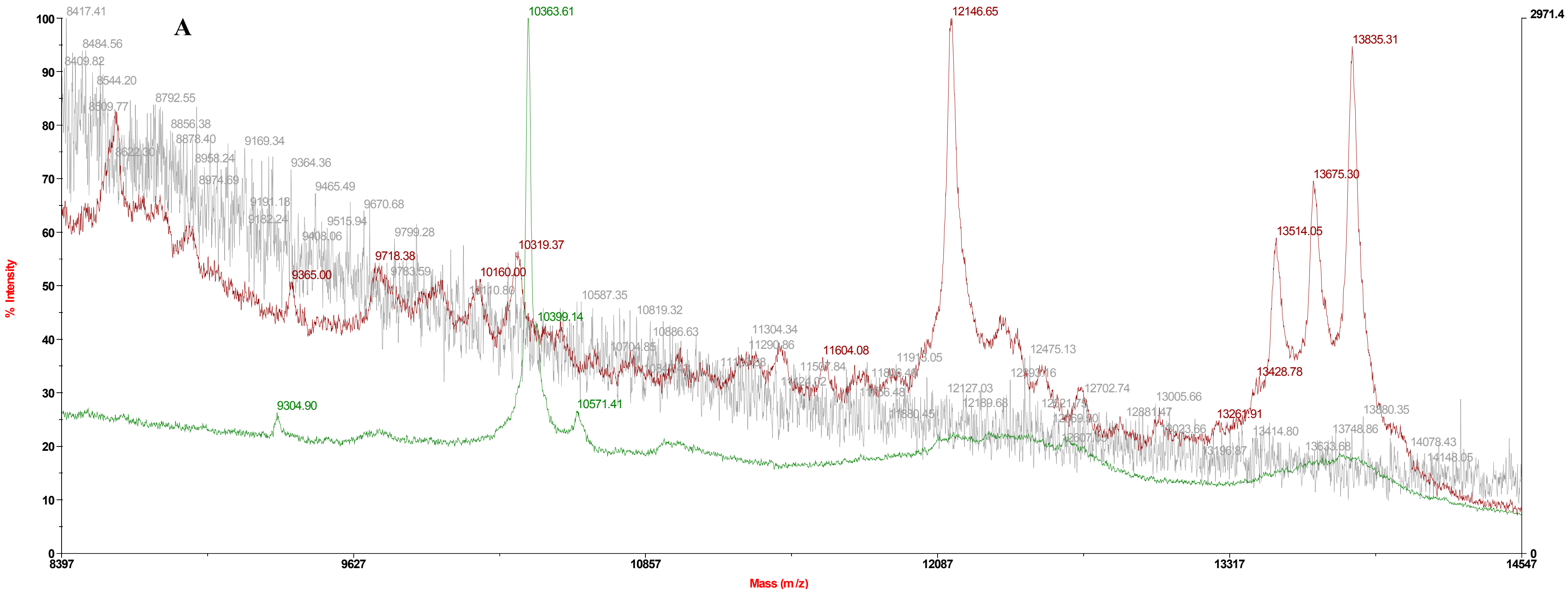

Figure S2. *Cont.*

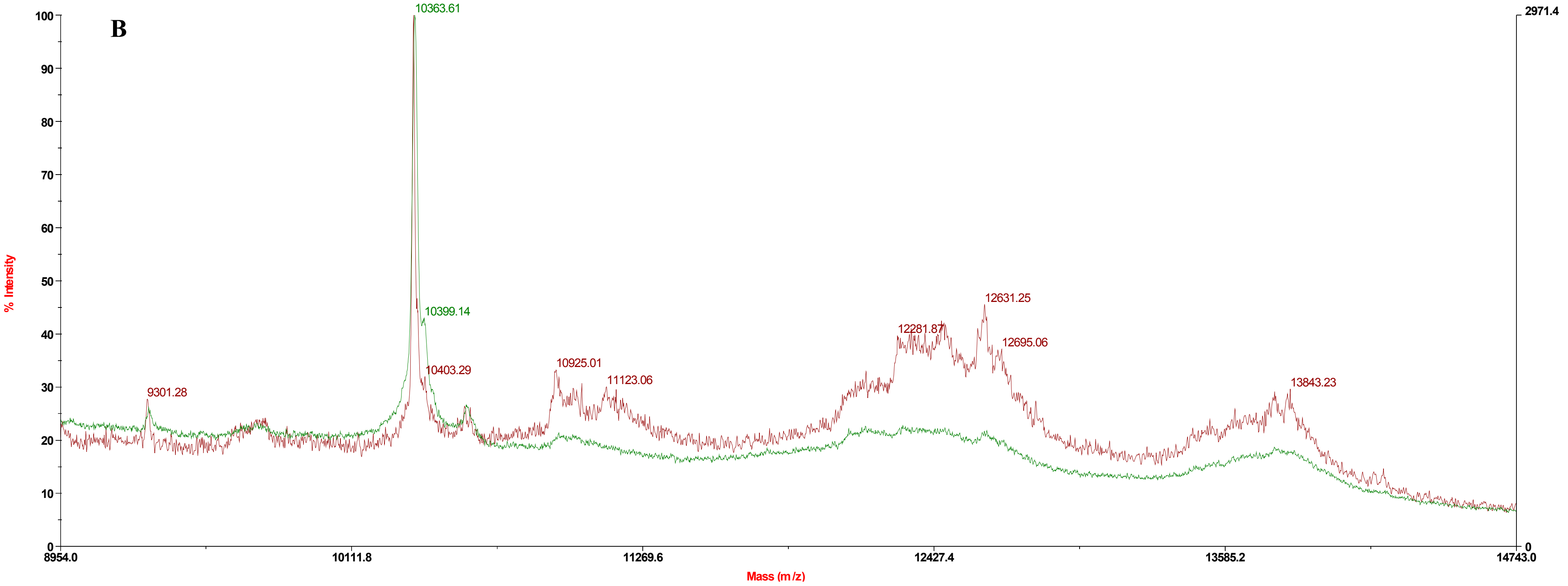

Figure S2. *Cont.*

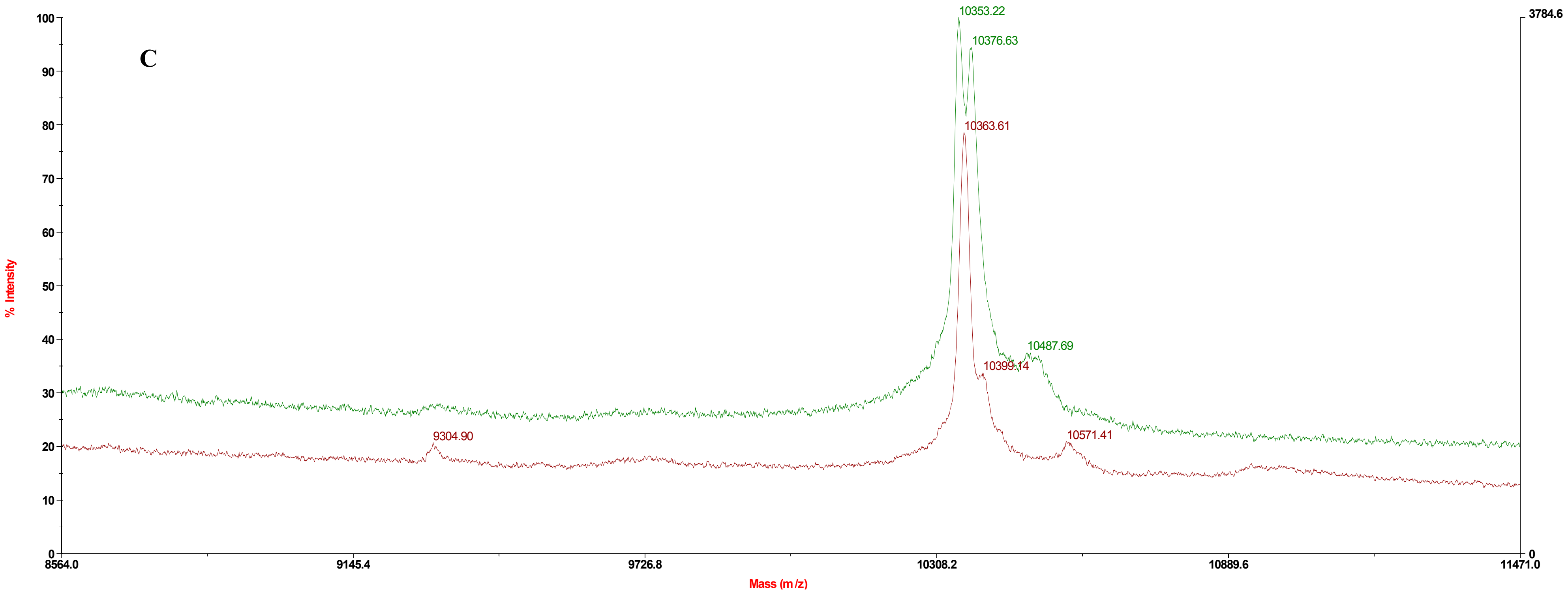

Figure S2. *Cont.*

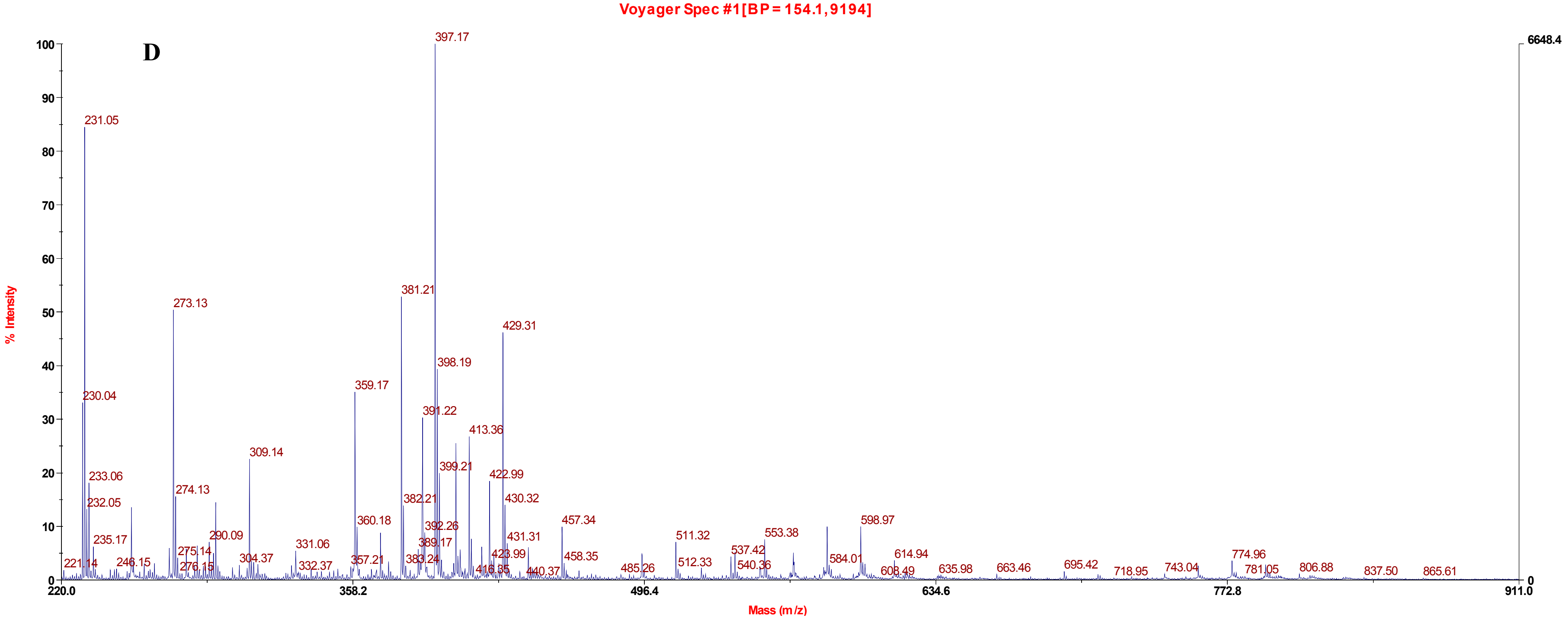

Table S1. MS/MS analysis of the CCF (**A**) and F1b fraction (**B**).

	Accession	Coverage	Peptides	AAs	MW [kDa]	Description
A	P02854	15.85	5	410	46.4	Provicilin precursor (Type B) (Fragment)-Pisum sativum (Garden pea)-[VCLB_PEA]
	P62926	37.69	5	130	13.9	Albumin-1 A precursor (PA1 A) [Contains: Albumin-1 A chain b (PA1b A) (Leginsulin A); Albumin-1 A chain a (PA1a A)]-Pisum sativum (Garden pea)-[ALB1A_PEA]
	P13918	6.75	2	459	52.2	Vicilin precursor-Pisum sativum (Garden pea)-[VCLC_PEA]
	P62929	37.69	5	130	13.9	Albumin-1 D precursor (PA1 D) (PsaA1b012) [Contains: Albumin-1 D chain b (PA1b D) (Leginsulin D); Albumin-1 D chain a (PA1a D)]-Pisum sativum (Garden pea)-[ALB1D_PEA]
	P62930	37.69	4	130	13.8	Albumin-1 E precursor (PA1 E) (PsaA1b014) (Psa) [Contains: Albumin-1 E chain b (PA1b E) (Leginsulin E); Albumin-1 E chain a (PA1a E)]-Pisum sativum (Garden pea)-[ALB1E_PEA]
	P15838	6.73	2	520	59.2	Legumin A2 precursor [Contains: Legumin A2 alpha chain (Legumin A2 acidic chain); Legumin A2 beta chain (Legumin A2 basic chain)]-Pisum sativum (Garden pea)-[LEGA2_PEA]
	P62931	16.15	2	130	13.9	Albumin-1 F precursor (PA1 F) (PsaA1b005/PsaA1b011) [Contains: Albumin-1 F chain b (PA1b F) (Leginsulin F); Albumin-1 F chain a (PA1a F)]-Pisum sativum (Garden pea)-[ALB1F_PEA]
	P05190	4.55	2	484	54.4	Legumin type B precursor [Contains: Legumin type B alpha chain (Legumin type B acidic chain); Legumin type B beta chain (Legumin type B basic chain)]-Vicia faba (Broad bean)-[LEGB4_VICFA]
	P14594	4.14	1	338	39.0	Legumin B [Contains: Legumin B alpha chain (Legumin B acidic chain); Legumin B beta chain (Legumin B basic chain)] (Fragment)-Pisum sativum (Garden pea)-[LEGB_PEA]
	P02856	19.35	2	124	14.0	Vicilin, 14 kDa component-Pisum sativum (Garden pea)-[VCL1_PEA]
	P05693	4.00	1	350	39.8	Legumin K [Contains: Legumin K alpha chain (Legumin K acidic chain); Legumin K beta chain (Legumin K basic chain)] (Fragment)-Pisum sativum (Garden pea)-[LEGK_PEA]
	P35699	9.18	3	98	10.4	Beta-elicitin MGM-beta-Phytophthora megasperma (Potato pink rot fungus)-[ELIB_PHYME]
	P24661	17.46	1	63	7.0	Bowman-Birk type proteinase inhibitor (FBI)-Vicia faba (Broad bean)-[IBB_VICFA]
	P04122	5.52	1	181	19.9	Lectin beta-1 and beta-2 chains-Lathyrus ochrus (Yellow-flowered pea)-[LECB_LATOC]
	P81651	8.79	1	91	9.2	Nonspecific lipid-transfer protein 1 (LTP 1) (Major allergen Pru ar 3)-Prunus armeniaca (Apricot)-[NLTP1_PRUAR]
	P09094	3.68	1	326	35.5	Glyceraldehyde-3-phosphate dehydrogenase, cytosolic (EC 1.2.1.12) (Fragment)-Nicotiana tabacum (Common tobacco)-[G3PC_TOBAC]

Table S1. *Cont.*

	Accession	Coverage	Peptides	AAs	MW [kDa]	Description
**B**	P02854	12.93	5	410	46.4	Provicilin precursor (Type B) (Fragment)-Pisum sativum (Garden pea)-[VCLB_PEA]
	O62823	10.28	2	214	24.3	Alpha-S1-casein precursor-Bubalus bubalis (Domestic water buffalo)-[CASA1_BUBBU]
	P13918	6.97	2	459	52.2	Vicilin precursor-Pisum sativum (Garden pea)-[VCLC_PEA]
	P62926	10.00	2	130	13.9	Albumin-1 A precursor (PA1 A) [Contains: Albumin-1 A chain b (PA1b A) (Leginsulin A); Albumin-1 A chain a (PA1a A)]-Pisum sativum (Garden pea)-[ALB1A_PEA]
	P02856	19.35	2	124	14.0	Vicilin, 14 kDa component-Pisum sativum (Garden pea)-[VCL1_PEA]
	Q12548	16.67	1	120	12.1	Superoxide dismutase [Cu-Zn] (EC 1.15.1.1) (Fragment)-Aspergillus japonicus-[SODC_ASPJA]
	P35699	9.18	3	98	10.4	Beta-elicitin MGM-beta-Phytophthora megasperma (Potato pink rot fungus)-[ELIB_PHYME]
	P02857	1.55	1	517	58.8	Legumin A precursor [Contains: Legumin A alpha chain (Legumin A acidic chain); Legumin A beta chain (Legumin A basic chain)]-Pisum sativum (Garden pea)-[LEGA_PEA]
	Q8D2Z7	1.47	1	475	54.6	UDP-N-acetylmuramate--L-alanine ligase (EC 6.3.2.8) (UDP-N-acetylmuramoyl-L-alanine synthetase)-Wigglesworthia glossinidia brevipalpis-[MURC_WIGBR]
	P02855	6.55	1	275	31.5	Provicilin (Type A) (Fragment)-Pisum sativum (Garden pea)-[VCLA_PEA]
